# Comparison of QIIME1 and QIIME2 for Analyzing Fungal Samples from Various Built Environments

**DOI:** 10.3390/microorganisms13112545

**Published:** 2025-11-06

**Authors:** Kensuke Watanabe, U Yanagi

**Affiliations:** 1 Graduate School of Engineering, Kogakuin University, Tokyo 163-8677, Japan; dd25003@g.kogakuin.jp; 2 School of Architecture, Kogakuin University, Tokyo 163-8677, Japan

**Keywords:** fungal microbiome, ITS2, NGS, Bioformatics, QIIME1, QIIME2, OTU, ASV, DADA2

## Abstract

This study evaluates the differences between bioinformatics pipelines by analyzing samples collected from various built environments. Previous comparative studies of microbial community analysis pipelines have largely focused on bacterial communities, mock communities, or soil fungi, often with small sample sizes, and have not specifically targeted built environments. Our results highlight key differences between OTU (QIIME1) and ASV (QIIME2) analyses. OTU analysis clusters OTUs at 97% similarity and tends to show higher diversity values in diversity analyses. Regarding abundantly detected fungi, OTU analysis identified more genera than ASV analysis. However, the OTU method has a high rate of false positives and false negatives, indicating low error-removal capability and suggesting that many fungal genera may have been detected. Therefore, a combined approach using OTU analysis combined with ASV analysis allows for both the comprehensive detection of dominant taxa and the inclusion of rare species. Overall, our findings emphasize that the choice of pipeline significantly influences the composition of the observed fungal community in built environments. Careful consideration of both OTU and ASV strategies can enhance the reliability and completeness of fungal metabarcoding studies, particularly when studying complex indoor microbial communities.

## 1. Introduction

Fungi comprise an extremely diverse group of organisms, including yeasts, molds, and mushrooms. It is estimated that millions of species exist on Earth, many of which remain undefined [1]. While fungi play an essential role in nature through the decomposition of organic matter and contribute to nutrient cycling, some species also exhibit pathogenicity toward humans, animals, and plants [2,3]. Particularly in human living environments, fungi can potentially cause diverse health impacts, such as acting as allergens [4], producing mycotoxins [5], and causing infections under conditions of immune suppression [6]. Clinically significant genera and species include *Aspergillus* (*A. fumigatus*, *A. flavus*, *A. niger*) [4,5,6], *Penicillium* (*P. chrysogenum*, *P. marneffei*) [5,6], *Cladosporium* [5], *Alternaria* [5], *Stachybotrys* (*S. chartarum*) [5], and *Fusarium* [5,6]. Among these species, *A. fumigatus* is the primary cause of fungal sensitization in asthma patients and the main causative agent of allergic bronchopulmonary aspergillosis (ABPA) [4]. Major fungal allergens include Asp f 1 Asp f 1, Asp f 2, Asp f 3, Asp f 4 Asp f 5, Asp f 6 derived from *A. fumigatus*, which can trigger asthma exacerbations and ABPA in sensitized individuals [4]. Up to 50% of severe asthma patients suffer from fungal sensitization, with approximately 6.5 million people worldwide estimated to have severe asthma with fungal sensitization (SAFS) [4]. Additionally, mycotoxins produced by indoor fungi include aflatoxins, ochratoxin A, trichothecenes, patulin, citrinin, and fumonisins, which cause health hazards such as carcinogenicity, immunosuppression, and renal/hepatic toxicity [5]. Aflatoxin B1 is known to be a potent carcinogen, whereas ochratoxin A is recognized for its nephrotoxicity [5]. These effects pose a particular threat to immunocompromised individuals and patients with chronic respiratory diseases, underscoring the importance of fungal control and proper management in indoor environments [4,6]. Accurately understanding the composition and diversity of fungal communities is therefore crucial from the perspectives of public health and environmental sanitation [7].

Fungal identification and analysis methods have traditionally relied on culture-based techniques and morphological observation. However, these approaches are often limited, particularly for species that are difficult to culture or for distinguishing morphologically similar taxa, making it challenging to fully assess fungal diversity. In recent years, advances in molecular biology techniques have established ribosomal DNA sequence analysis as the primary method for fungal identification [8]. Among these analyses, the Internal Transcribed Spacer (ITS) region has been internationally adopted as the standard fungal DNA barcode, with the ITS1 and ITS2 regions serving as the principal targets for metabarcoding analysis [9,10]. With the widespread application of Next-Generation Sequencing (NGS), comprehensive analyses of fungal communities based on these ITS regions have rapidly expanded, revealing the presence of diverse fungi previously undetectable by means of culture methods [11].

Research on fungal communities in built environments such as homes, hospitals, and schools has gained particular attention in recent years [12,13]. Indoor fungal exposure has been reported to be associated with the onset and exacerbation of allergic rhinitis and asthma, in addition to respiratory diseases such as hypersensitivity pneumonia. Thus, analyzing indoor fungal communities is of considerable importance in both environmental epidemiology and clinical medicine [14,15,16]. However, their composition is influenced by a variety of factors, including geographical conditions [17], seasonal variations [18,19], building materials, and ventilation systems [20,21]. In addition, outcomes may vary based on differences in analytical methods and bioinformatics approaches [22,23]. As a result, direct comparison and integration of data across studies remain challenging [24]. Furthermore, with regard to quantitative standards for fungal exposure, the WHO guidelines do not specify quantitative exposure limits such as spore counts or CFU/m^3^. They clearly state that there is insufficient scientific evidence to establish numerical standards for biological contaminants [25]. Similarly, the EPA guidelines do not define quantitative concentration limits for fungal exposure. Instead, they provide recommendations focusing on moisture and mold control, humidity management, and improved ventilation [26].

To conduct fungal community analysis using NGS data, a bioinformatics pipeline is essential to perform a series of computational steps, including sequence quality control, taxonomic assignment, and diversity metric calculation [27,28,29]. Among the available platforms, QIIME (Quantitative Insights Into Microbial Ecology) is one of the most widely used internationally [30,31,32]. QIIME1, released in 2010, employs an OTU (Operational Taxonomic Unit) clustering approach based on 97% sequence similarity [33,34] and has been applied to the analysis of diverse microbial communities, including bacteria and fungi [33,34,35]. In contrast, QIIME2, which has gained widespread adoption since 2018 [36], enables ASV (Amplicon Sequence Variant)-based processing through tools such as DADA2 and Deblur [37,38,39]. It incorporates multiple improvements, including enhanced sequence error correction accuracy, improved reproducibility, and extensibility, through its plugin-based architecture [28,40].

Mock communities (artificial microbial communities) composed of known species are frequently employed in performance evaluation and comparative studies of analysis pipelines [41,42,43]. They are particularly useful for evaluating taxonomic identification accuracy and sequence processing algorithms [44] and have been widely applied in bacterial 16S analysis [45]. However, the use of mock communities in fungal ITS analysis presents several challenges. First, the ITS region exhibits substantial variation in length and base composition both between and within species [46,47]. This variation results in differences in PCR amplification efficiency and sequence coverage among species, often causing the observed sequence ratios to deviate from the expected community composition [44]. Second, fungal ITS reference databases are less comprehensive than bacterial 16S databases [30,48]. For example, while the UNITE database currently contains approximately 3.8 × 10^6^ ITS sequences and roughly 2.4 × 10^5^ Species Hypotheses, the bacterial 16S reference database (SILVA) contains millions of SSU sequences covering a broader range of taxa. Therefore, even known species included in mock communities may not always be accurately assigned at all taxonomic levels [49,50]. Moreover, the availability of standardized fungal mock communities is limited, making it difficult for researchers to conduct evaluations under identical conditions [51,52]. Collectively, these factors complicate the interpretation of results in pipeline comparisons and may introduce biases different from those observed in real environmental samples [38,45].

Comparative studies between QIIME1 and QIIME2 have been widely reported for bacterial 16S rRNA gene analysis [32,53,54], demonstrating that ASV-based QIIME2 provides higher reproducibility and resolution [55,56]. In contrast, direct comparative studies of QIIME1 and QIIME2 targeting fungal ITS regions are extremely limited [38,57,58], and the extent to which differences in analysis pipelines influence results, particularly in the fungal community analysis of residential environments, remains insufficiently validated [58,59]. Given the substantial diversity of the fungal-specific ITS region [46,60], the incompleteness of fungal reference databases [30], and the challenges associated with using mock communities [38,59], the impact of analysis method choice on results may be even greater for fungi than for bacteria [38,57,59].

Fungal communities are known to vary depending on building type and differences in air conditioning and ventilation systems, with fungal community composition differing between indoor and outdoor environments. Furthermore, bioinformatics pipelines have been suggested to potentially influence the results of fungal community analysis. Based on the above findings, we propose the following three hypotheses:

(1) Indoor fungal communities differ depending on building type and variations in air conditioning and ventilation systems.
(2) The composition of fungal communities differs between indoor and outdoor environments.
(3) Differences are observed in the analysis results obtained using QIIME1 and QIIME2.

In this study, we aimed to compare fungal community analyses conducted with QIIME1 and QIIME2 using identical ITS sequence datasets obtained from various built environments, evaluating the outcomes in terms of diversity metrics and taxonomic composition. To achieve this goal, fungal communities were analyzed in various built environments across humid temperate and subtropical regions of Japan, including housing, office buildings, and movie theaters. Samples were collected from the air, surfaces, and settled dust in order to capture the diversity of indoor microbiomes.

## 2. Materials and Methods

### 2.1. Data Acquisition Source

The dataset was measured across groups (outdoors in summer, outdoors in winter, an office in summer, an office in winter, a movie theater, an air conditioner filter, and an air conditioner coil), totaling 84 samples (Table 1).

In the summer of 2019 and winter of 2020, a total of 32 samples were collected, comprising 8 samples from each group. Sampling was performed by attaching filters (pore size 0.3 μm) to air pumps in five office buildings in Tokyo and three in Aichi Prefecture, in addition to at their outdoor locations. The filters exhibit high capture efficiency of approximately 99.7 to 99.99% for 0.3 μm particles and demonstrate high performance for particles ranging from 0.1 μm to several micrometers. The primary particle capture mechanisms include diffusion, inertial impaction, and interception [61]. Each sample was collected at a flow rate of 3 L/min for 60 min, corresponding to a total volume of 180 L.

In the summer of 2023, a total of 18 samples were collected from the front and rear floor surfaces after three screenings at each of two movie theaters in Tokyo and one in Chiba Prefecture. Sterile cotton swabs were used to sample 10 cm × 10 cm areas located directly beneath seats, avoiding aisles. Each swab was stored individually in a sterile container (ST-25, ELMEX, Tokyo, Japan) containing 10 mL of phosphate-buffered saline (PBS) in a freezer (−20 °C) and subsequently processed in the laboratory.

In the summer of 2021, a total of 34 fungal samples were collected from 17 residential air conditioners in Kanagawa Prefecture—17 samples each from the filters and coil surfaces. Sterile swabs were used to wipe fungi from a 25 cm^2^ surface area of each component. The swabs were then placed individually in containers (ST-25, ELMEX) with 10 mL of sterile PBS in a freezer (−20 °C) and processed in the laboratory. For each component, samples were collected from two sites, corresponding to a total sampled area of 50 cm^2^.

### 2.2. DNA Extraction—ITS Sequencing

After the fungal samples adhering to surfaces were collected as described above, the swabs were processed using a Stomacher (Stomacher^®^ 80 Biomaster, seward, West Sussex, UK). A mixture of 3 mL DNase-free water and 2 mL sample solution was prepared, and DNA extraction was performed with the Stomacher. The processed sample was then removed from the Stomacher bag, transferred to a 1.5 mL microcentrifuge tube, and centrifuged at 4 °C and 3000 rpm for 30 min using a KUBOTA5911 centrifuge (KUBOTA, Tokyo, Japan) to pellet the fungal material. DNA was subsequently purified using the NucleoSpin^®^ Tissue Kit (740952, MACHEREY-NAGEL, Düren, Germany). During the purification process, the solution was vortexed, heated, mixed with ethanol, centrifuged, and subjected to additional standard processing steps according to the manufacturer’s protocol.

For each sample, the fungal ITS2rRNA gene was amplified using primers “5′-ACACTCTTTCCCTACACGACGCTCTTCCGATCT-GTGAATCATCGARTCTTTG-3′ (gITS7)” and “5′-GTGACTGGAGTTCAGACGTGTGCTCTTCCGATCT-TCCTCCGCTTATTGATATGC-3′ (ITS4)”. DNA amplification and ITS2 rRNA gene sequencing were performed using the Illumina NGS platform, with DNA processing outsourced to a commercial laboratory.

DNA quality was verified using the Agilent 2200 TapeStation (Agilent, Santa Clara, CA, USA), and all samples containing nucleic acid concentrations of sufficient quality, and only samples with nucleic acid concentrations meeting quality and quantity thresholds, were processed. The sequencing libraries were pooled, and PCR products were purified using AMPure XP beads (Beckman Coulter, San Jose, CA, USA) (1:1 bead-to-sample volume ratio) to enhance library quality.

### 2.3. OTU Sequence Assignment

Fungal OTUs were generated using QIIME1 (1.9.1). Reads with a quality score of ≤20 were removed, while no trimming of read length was applied (Table 2). Chimeric sequences were removed, and taxonomic assignment was performed using UNITE version 10 (19 February 2025). In QIIME1, operational taxonomic units (OTUs) were clustered using the 97% similarity threshold, which has been widely adopted in microbial ecology research. This threshold was originally derived from studies of the bacterial 16S rRNA gene and is based on the empirical criterion that sequences sharing 97% or greater similarity can generally be considered members of the same species [62]. Although the fungal ITS region exhibits greater variability than 16S rRNA, the same 97% threshold has been used for convenience to maintain consistency with past studies and comparability between datasets. We also adopted the 97% threshold.

### 2.4. ASV Sequence Determination

Fungal ASVs were generated using QIIME2 (2025.4). To perform quality filtering, the DADA2 parameters --p-max-ee-f and --p-max-ee-r were applied with the default value of 2.0 (Table 2). No unique trimming was performed; instead, read lengths that maximized the number of retained reads in DADA2 were used. Specifically, the parameters --p-trunc-len-f and --p-trunc-len-r were set to 160 (forward) and 150 (reverse), respectively. Taxonomic assignment was conducted using UNITE version 10 (19 February 2025).

### 2.5. Statistical Analysis Methods

In this study, the Wilcoxon signed-rank test was performed using IBM SPSS Statistics version 29 to compare taxonomic hierarchy and α diversity values of fungal communities between QIIME1 and QIIME2. Mantel test analyses were performed using R Statistical Software (v4.2.0), via the vegan R package (v2.6-4) to compare taxonomic hierarchy, β diversity values of fungal communities.

The Wilcoxon signed-rank test is a paired nonparametric test, and paired values were obtained from QIIME1 and QIIME2 analyses of the same samples. A *p*-value of <0.05 was considered statistically significant. For comparisons of taxonomic hierarchies, relative abundance was used. α diversity was assessed with three metrics: The observed species index, Shannon index, and PD whole tree index. To ensure robustness, a fixed sequencing depth (sample depth) was randomly resampled 10 times from each sample, and diversity indices were calculated for each iteration. The median value from the 10 obtained values for each metric was adopted as the representative metric for subsequent statistical testing. This approach provides more stable representative values while accounting for variation due to resampling. β diversity was evaluated using both weighted and unweighted UniFrac distance, Bray–Curtis distances, and Jaccard distances calculated by each pipeline.

## 3. Results

### 3.1. Microbial Community α and β Diversity

To evaluate differences between pipelines, we compared α and β diversity generated by QIIME1 and QIIME2. Statistical significance of α diversity was evaluated using the Wilcoxon signed-rank test. *: *p* < 0.05 and **: *p* < 0.001. Statistical significance of β diversity was evaluated using the Mantel test. *: *p* < 0.05, **: *p* < 0.01, and ***: *p* < 0.001.

**Figure 1:**
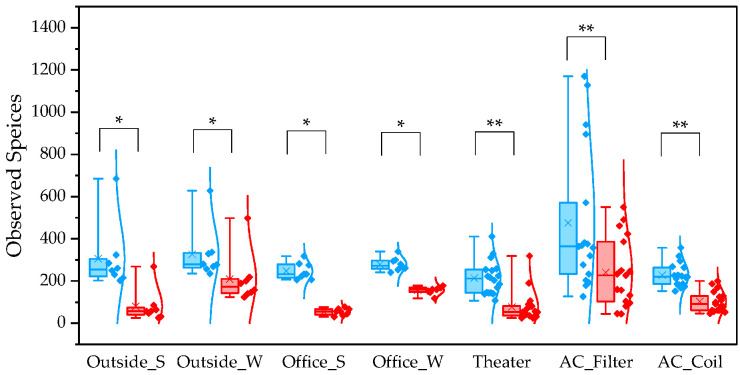
The quartile values of the observed species index (Blue: QIIME1; Red: QIIME2). *: *p* < 0.05, **: *p* < 0.001.

**Figure 2:**
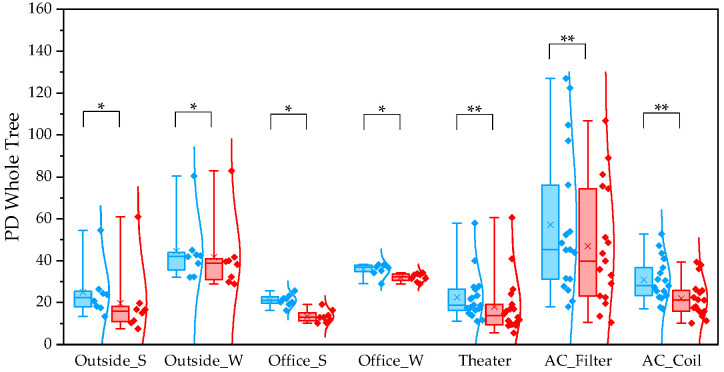
The quartile values of the PD whole tree index (Blue: QIIME1; Red: QIIME2). *: *p* < 0.05, **: *p* < 0.001.

**Figure 3:**
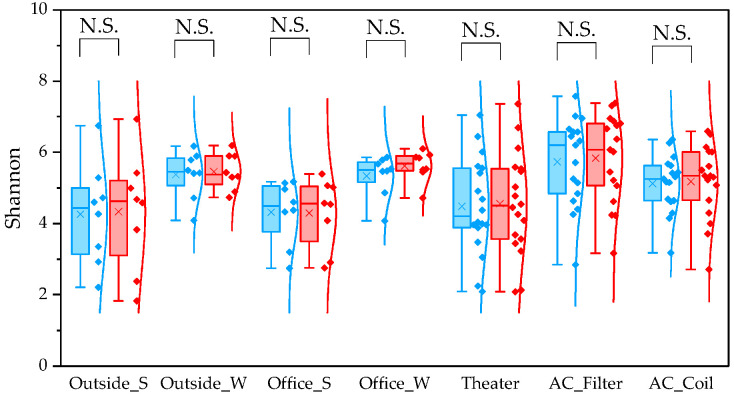
The quartile values of the Shannon index (Blue: QIIME1; Red: QIIME2). N.S.: Not Significant.

For the observed species index, QIIME1 yielded significantly higher values than QIIME2 across all sample types (Figure 1). Variability was also lower in QIIME2, particularly in winter outdoor samples. For the PD whole tree index, QIIME1 likewise produced significantly higher values across all sample types (Figure 2). In contrast, the Shannon index showed no significant difference between the two pipelines (Figure 3). When comparing sampling methods and applications, the observed spices index and PD whole tree index indicated that indoor office air samples exhibited less variability than outdoor air samples in both pipelines, suggesting that indoor air quality is relatively consistent across building types. The Shannon index revealed no clear discernible difference in variability between indoor and outdoor air; however, seasonal patterns were evident, with lower variability observed in winter. Similarly, differences emerged between air conditioner filters and coils: for the observed species and PD whole tree indices, coils exhibited lower variability than filters.

**Figure 4:**
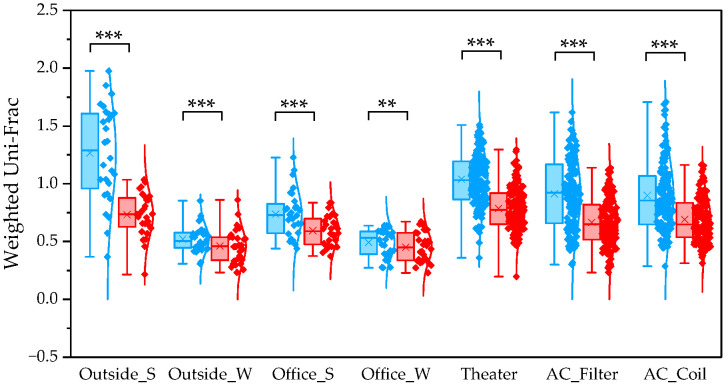
The quartile values of Weighted UniFrac (Blue: QIIME1; Red: QIIME2). **: *p* < 0.01, and ***: *p* < 0.001.

**Figure 5:**
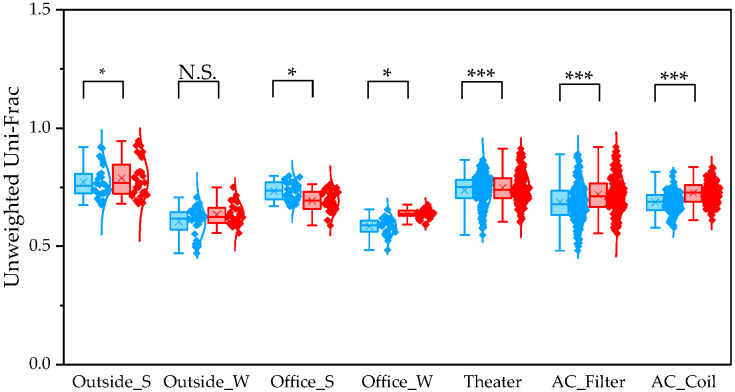
The quartile values of Unweighted UniFrac (Blue: QIIME1; Red: QIIME2). *: *p* < 0.05, and ***: *p* < 0.001, N.S.: Not Significant.

**Figure 6:**
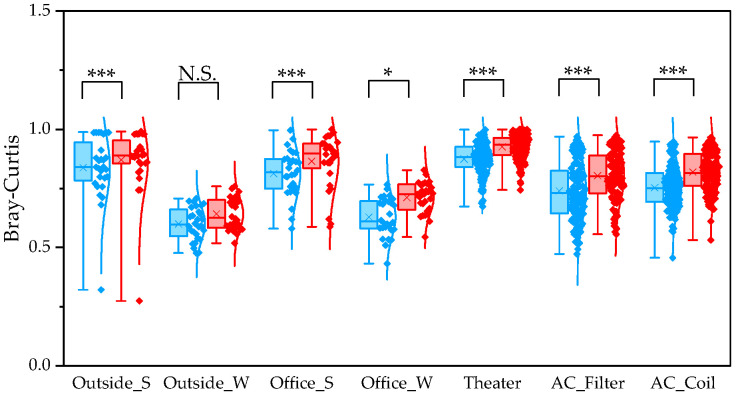
The quartile values of Bray–Curtis distance (Blue: QIIME1; Red: QIIME2). *: *p* < 0.05, and ***: *p* < 0.001, N.S.: Not Significant.

**Figure 7:**
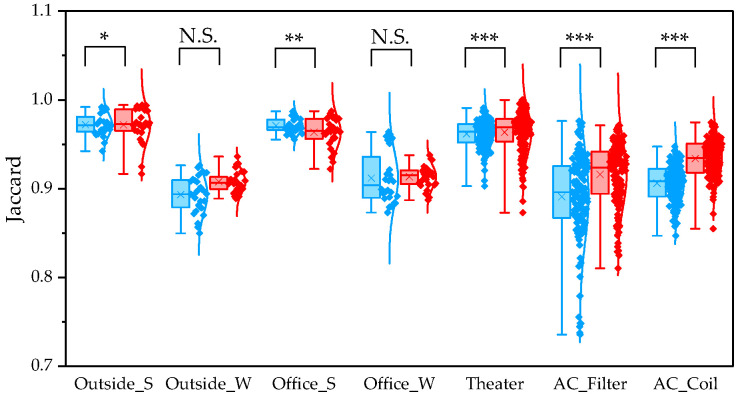
The quartile values of Jaccard distance (Blue: QIIME1; Red: QIIME2). *: *p* < 0.05, **: *p* < 0.01, and ***: *p* < 0.001, N.S.: Not Significant.

To determine β diversity, pipelines were compared using Weighted and Unweighted UniFrac distances. Weighted UniFrac distances were significantly higher in QIIME1 than in QIIME2 across all sample types (Figure 4). Unweighted UniFrac distances also showed significant differences between the two pipelines, except for winter outdoor samples (Figure 5). Regarding Bray–Curtis distance, significant differences were observed between the two pipelines for all sample types except the winter outdoor sample (Figure 6). Regarding Jaccard distance, significant differences were observed between the two pipelines for all sample types except the winter outdoor and office samples (Figure 7).

### 3.2. Taxonomic Differences Related to the Pipeline

We compared differences in the number of OTUs and ASVs obtained after base sequence determination (Table 3). Sequencing depth was confirmed using a rarefaction curve. Sequence analysis yielded 130,977 ± 18,076 reads from the summer outdoor sample, 76,097 ± 8832 reads from the winter outdoor sample, 125,850 ± 20,762 reads from the summer office sample, 76,714 ± 10,179 reads from the winter office sample, 96,637 ± 18,260 reads from the movie theater sample, 28,824 ± 7229 reads from the air conditioner filter sample, and 27,349 ± 5948 reads from the air conditioner coil sample. After processing with QIIME1 and QIIME2 (DADA2), QIIME1 consistently produced higher values; in comparison, QIIME2 yielded lower counts and diversity for ASVs. In terms of base-call reduction rates during sequence analysis, QIIME1 showed reductions of up to 75% in some summer outdoor samples, whereas QIIME2 showed reductions of up to 42% in some winter office samples, reflecting differences in filtering stringency between the pipelines. Overall, significant differences were observed in OTU and ASV counts, and when focusing on specific groups, the average difference was at least twofold.

**Figure 8:**
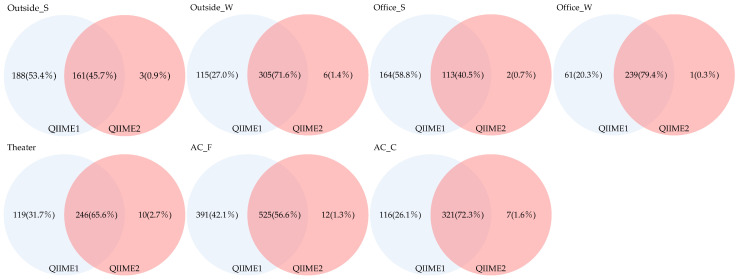
Venn diagram of genera detected across different pipelines.

The Venn diagram presented in Figure 8 illustrates the number of genera detected commonly across pipelines and those detected exclusively by each. The proportion of shared genera across sample types ranged from 40.5% to 79.4%, indicating relatively few overlapping genera. Genera detected exclusively using QIIME1 accounted for 20.3–58.8%; in comparison, those detected only by QIIME2 accounted for 0.3–2.7%, demonstrating that QIIME1 detected a greater number of genera.

**Figure 9:**
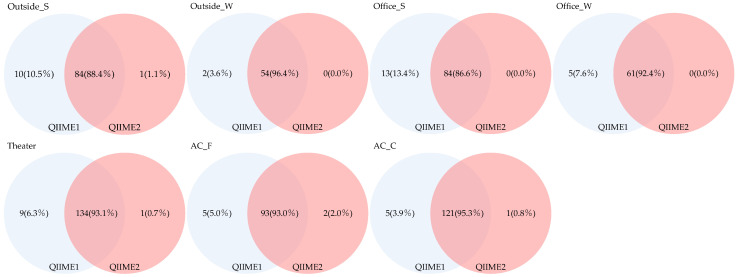
Venn diagram of genera detected with a relative abundance of 1% or higher across different pipelines.

Furthermore, we analyzed genera restricted to those with a relative abundance of ≥1% (Figure 9). The proportion of genera detected across all sample types ranged from 86.6% to 96.4%, indicating that relatively few genera were shared among all types. Genera detected exclusively using QIIME1 accounted for 3.6–13.4%; in comparison, those detected only by QIIME2 accounted for 0.0–2.0%, suggesting that QIIME1 detected more genera. In contrast, when genera with relative abundances below 1% were considered, significant differences between the two pipelines became apparent.

We also examined differences related to sampling methods and applications. Seasonal variation was observed in the number and proportion of fungal genera shared between indoor and outdoor samples, with more genera commonly detected in winter. Differences were likewise noted between air-conditioner filters and coils, with coils showing higher numbers of shared genera. When restricting the analysis to genera with a relative abundance of ≥1%, coils still exhibited higher numbers than filters, although the proportions were comparable.

### 3.3. Relative Composition Ratios

**Figure 10:**
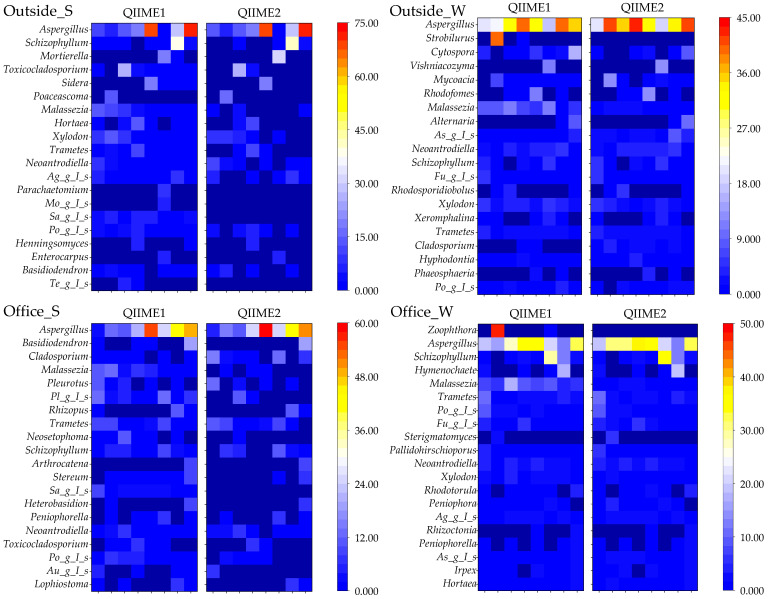

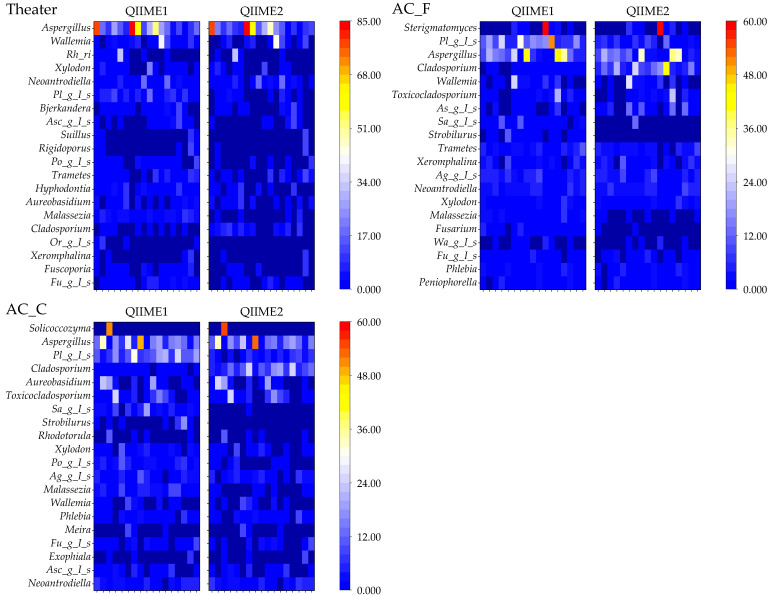
Top 20 fungal genera detected using different pipelines. (*Ag_g_I_s*: *Agaricomycetes_gen_Incertae_sedis*, *Mo_g_I_s*: *Mortierellaceae_gen_Incertae_sedis*, *Sa_g_I_s*: *Saccharomycetales_gen_Incertae_sedis*, *Po_g_I_s*: *Polyporaceae_gen_Incertae_sedis*, *Te_g_I_s*: *Teratosphaeriaceae_gen_Incertae_sedis*, *As_g_I_s*: *Aspergillaceae_gen_Incertae_sedis*, *Fu_g_I_s*: *Fungi_gen_Incertae_sedis*, *Pl_g_I_s*: *Pleosporales_gen_Incertae_sedis*, *Au_g_I_s*: *Auriculariales_gen_Incertae_sedis*, *Rh_ri*: *Rhodosporidiobolus*, *Asc_g_I_s*: *Ascomycota_gen_Incertae_sedis*, *Or_g_I_s*: *Orbiliaceae_gen_Incertae_sedis*, and *Wa_g_I_s*: *Wallemiaceae_gen_Incertae_sedis*).

Differences between pipelines were examined to determine the top 20 genera with the highest relative abundances (Figure 10).

Several genera, including *Parachaetomium*, *Mortierellaceae_gen_Incertae_sedis*, *Saccharomycetales_gen_Incertae_sedis*, *Teratosphaeriaceae_gen_Incertae_sedis*, *Strobilurus*, *Zoophthora*, and *Orbiliaceae_gen_Incertae_sedis*, were not detected using QIIME2. Differences were also noted for genera that were abundantly detected but remain unresolved at the genus level.

Among all detected genera, *Aspergillus* was the most prevalent, ranking third or higher across all sample types. Although some groups exhibited significant differences between the two pipelines, the overall results were relatively consistent. Notably, the genera *Aspergillus*, *Malassezia*, *Trametes*, and *Schizophylum* include fungal species known to cause disease.

## 4. Discussion

Metabarcoding has become an indispensable tool for microbial community analysis, but the resulting data is vast, making bioinformatics analysis challenging. Selecting an appropriate pipeline is crucial for establishing analysis protocols.

In this study, we compared QIIME1 (97% threshold) for OTU analysis and QIIME2 (DADA2) for ASV analysis using a total of 84 samples from built environment microbiomes: summer and winter outdoor air, summer and winter indoor office air, cinema floor surface fungi, and residential air conditioner filter and coil fungi. QIIME1, frequently used until around 2020, has received reduced attention since the release of QIIME2 in 2018. However, the authors of studies comparing bacterial community analysis pipelines have reported that when the goal is “screening for rare or low-frequency candidate taxa,” QIIME1 may perform better than other methods in approaches that tolerate an increased potential for false positives while combining subsequent rigorous validation. The above demonstrates that new analysis methods are not always optimal and highlights the importance of selecting the appropriate tool based on research objectives [32,53,63,64,65]. However, the results of studies involving the use of mock communities have shown that QIIME1 (uclust) tends to generate spurious OTUs and assign a large portion of total counts to them [53]. Furthermore, QIIME1 tends to have a relatively high false positive rate and a low false negative rate; in comparison, QIIME2 (DADA2) exhibits a lower false positive rate and a slightly higher false negative rate. In studies targeting bacteria, the characteristics of each pipeline have been broadly confirmed [66].

In this study, we graphically represented and analyzed differences in α diversity, β diversity, read counts, number of detected genera, and relative abundance. As a result, a difference was confirmed between QIMME1 and QIMME2, consistent with previous studies.

The results showed significant differences between the pipelines in most analyses except for the Shannon index. The difference in the number of filtered reads was significant, and this result was reflected in the observed species. This finding is thought to be strongly influenced by clustering and filtering in ASV analysis, consistent with previous studies. QIIME1 outputs a very large number of OTUs, most of which are estimated to be spurious [53], a factor that is believed to have similarly manifested in this study. This finding was thought to be strongly influenced by the effects of clustering and filtering in ASV analysis. Furthermore, the PD whole tree value was lower for QIIME2, likely due to the reduction in species number. No significant differences were observed in the Shannon index across all samples. The lack of significance in the Shannon index suggests that the abundance and evenness contributing at high frequencies are nearly identical between the two pipelines. However, differences in the abundance of low-frequency species and the presence of numerous false positives are indicated. Previous studies have noted that differences exist between pipelines, suggesting it is more appropriate to focus on qualitative differences between samples rather than quantitative values [67,68]. These differences likely exist because ASV analysis removes many rare bacterial species, and since the Shannon index measures community evenness, removing species with inherently low relative abundances does not alter the index value. Furthermore, when focusing on differences in sampling methods and applications, we found that variations in the observed species and PD whole tree indices differed between office and outdoor airborne samples. Outdoor air is subject to numerous external factors such as temperature, humidity, wind direction, and rainfall, with conditions changing hourly. In contrast, Japanese office spaces are regulated by the Act on Maintenance of Sanitation in Buildings to maintain specified temperature and humidity ranges, likely resulting in less variation in fungal communities. However, while no difference was observed between offices and outdoors for the Shannon index, a difference was seen between summer and winter. This difference is thought to be because winter is characterized by low temperature and humidity, creating an environment unsuitable for fungal growth. Consequently, only a limited number of fungi grow during this time period, resulting in a significantly different microbial community balance. Furthermore, focusing on the coils and filters of residential air conditioners, we found that filters showed higher values for all three indices. Compared to coils, filters harbored a greater number of observed species, exhibited greater phylogenetic diversity, and demonstrated higher evenness. During cooling operation, the humidity inside air conditioners is known to be higher than that of the indoor air [69], creating an environment conducive to fungal growth. In contrast, filters are located relatively close to the indoor environment and are easily influenced by outdoor air entering through open windows or ventilation systems. However, the coils are situated within the confined space of the unit, which makes it difficult for spores and various fungi originating from outdoor air to enter. Therefore, the α-diversity observed on the coils was considered to be lower than that observed on the filters. Previous bacterial studies have shown that the Shannon index is higher on filters than on coils. Since bacteria, like fungi, tend to thrive in warm and humid environments, this finding suggests that a similar difference may also exist for fungi [69].

In this study, we utilized the UniFrac distance, analyzed based on species phylogenetic trees. For Weighted UniFrac, QIIME2 diversity indices were found to be significantly lower across all sample groups. As mentioned above, ASV analysis that excludes low-frequency species is a denoising method. Since it performs misalignment estimation and correction, it tends to suppress the false positive rate. However, it has been noted that sensitivity (the possibility of missing true sequences) may be slightly reduced, potentially leading to an underestimation of the diversity of rare bacterial species [70].

**Figure 11:**
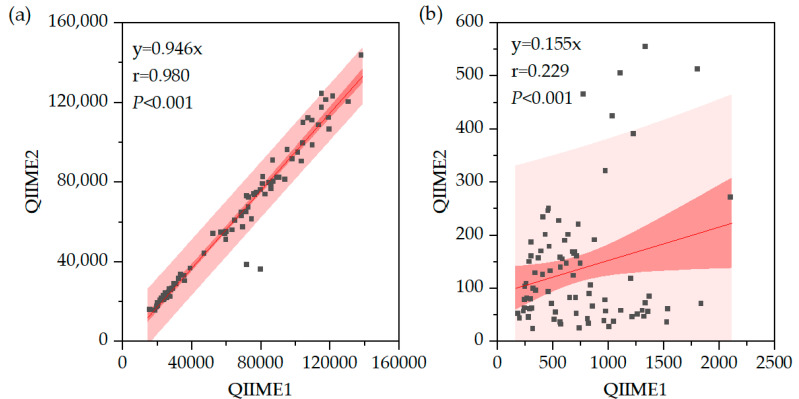
(**a**) Correlation of Libraries in QIIME1 and QIIME2. (**b**) Correlation of OTUs/ASVs in QIIME1 and QIIME2.

Comparison of read counts revealed no significant difference in the number of libraries but a significant difference between OTUs and ASVs, suggesting that clustering and filtering effects are substantial in ASV analysis (Figure 11). The 95% confidence intervals and 95% prediction intervals are shown in Figure 11. Statistical significance was evaluated using the Wilcoxon signed-rank test. This difference was more than sixfold, indicating a large disparity between OTUs and ASVs analyses, highlighting the importance of pipeline selection (Figure 11b). Furthermore, the range of OTU values relative to raw library sizes showed a smaller difference between minimum and maximum values compared to ASVs, indicating differences in sensitivity to clustering and filtering between samples.

In the Venn diagram analysis, fungi detected identified to the genus level and those additionally detected with a relative abundance of 1% or higher were analyzed based on the number of detections. In the analysis covering all fungal genera, although there were differences in absolute sample numbers between sample groups, the proportion of fungi uniquely detected using QIIME2 was lower compared to QIIME1. In the analysis restricted to relative abundance ≥1%, the proportion of fungal species detected only by QIIME1 was smaller compared to the previous analysis. The results of this analysis also revealed that pipeline differences were significant for fungi with low relative abundance.

To further deepen the analysis, we presented heatmaps showing the top 20 most abundant fungal genera detected in each sample. Furthermore, some fungal genera were detected only with QIIME1, whereas several genera, including unassigned taxa, were not detected with QIIME2. This finding suggests that differences in reference databases and classification methods influence the analysis results. Therefore, strong pipeline dependency exists even at the genus level, necessitating caution in interpreting results, particularly in fungal community analysis [65]. Among the detected genera, *Aspergillus* was prevalent overall. In summer, regarding airborne fungal samples collected in offices and outdoors, the results suggest that outdoor influences also affect indoor environments.

## 5. Conclusions

In this study, we analyzed differences between pipelines using samples collected from various built environments. Previous comparative studies of microbial community analysis pipelines have focused on bacterial communities, mock communities, or soil fungi, but not in built environments, and the number of samples compared is also small in such studies. In this regard, this study is novel, and the findings are summarized below.

(1) There are differences between OTU analysis and ASV analysis. Since OTU analysis involves OTU clustering at 97% similarity, sequence errors and clustering-induced partitioning can increase the apparent number of taxa, particularly affecting rare fungi. Therefore, when performing diversity analyses, OTU analysis tends to yield higher diversity values.
(2) Regarding abundantly detected fungi, OTU analysis detects a greater number thereof; however, caution is warranted, as this study also suggested the possibility of false positives.
(3) *Aspergillus* was predominantly detected across all groups, irrespective of pipeline or building application.
(4) The α diversity index was higher in outdoor air than in office interiors, and the air conditioning filters exhibited greater α diversity than coils. These findings indicate an environment conducive to the growth of numerous fungi.

## Figures and Tables

**Table 1:** Summary of measurement targets.

Sample Type	Sample ID	Number of Samples	Year	Way	Time Until Sequence (Month)
outdoors in summer	Outside_S	5 (Tokyo), 3 (Aichi prefecture)	2019	Air sample 3 L/min (60 min)	3
outdoors in winter	Outside_W	5 (Tokyo), 3 (Aichi prefecture)	2020	Air sample 3 L/min (60 min)	8
office in summer	Office_S	5 (Tokyo), 3 (Aichi prefecture)	2019	Air sample 3 L/min (60 min)	3
office in winter	Office_W	5 (Tokyo), 3 (Aichi prefecture)	2020	Air sample 3 L/min (60 min)	8
movie theater	Theater	18	2023	Swab 100 cm^2^	3
air conditioner filter	AC_Filter	17	2021	Swab 50 cm^2^	4
air conditioner coil	AC_Coil	17	2021	Swab 50 cm^2^	4

**Table 2:** Schematic diagram comparing the two pipelines.

Pipeline	QIIME1		QIIME2	
Primer trimming	cutadapt		qiime cutadapt trim-paired	
	--errors	0.2	--p-error-rate	0.2
	--trimmed-only	TRUE	--p-discard-untrimmed	TRUE
Quality filtering	cutadapt			dada2 --p-max-ee-f/ --p-max-ee-r
	--quality-cutoff	20		
	--minimum-length	2		
Pooling mode	NGmerge		qiime dada2 denoise-paired	
	-m: Minimum overlap of the paired-end reads	20	--p-trunc-len-f	190
	-e: Minimum overlap of dovetailed alignments	100	--p-trunc-len-r	180
			--p-trim-left-f	0
			--p-trim-left-r	0
			--p-max-ee-f	2
			--p-max-ee-r	2
			--p-pooling-method	default ‘independent’
Chimera handling	usearch		--p-chimera-method	default ‘consensus’
	-strand	plus		
Classifier training	pick_de_novo_otus.py		qiime feature-classifier classify-sklearn	
	default		default	
Rarefaction depth	alpha_rarefaction.py		qiime diversity alpha-rarefaction	
	--parameter_fp	Minimum lead count	--p-sampling-depth	Minimum lead count
	beta_diversity_through_plots.py		qiime diversity core-metrics-phylogenetic	
	default		--p-max-depth	Maximum number of leads

**Table 3:** Output data after processing sequence data with different pipelines.

	Outside_S		Outside_W		Office_S		Office_W		Theater		AC_Filter		AC_Coil	
	QIIME1	QIIME2	QIIME1	QIIME2	QIIME1	QIIME2	QIIME1	QIIME2	QIIME1	QIIME2	QIIME1	QIIME2	QIIME1	QIIME2
Raw	130,977 ± 18,076		76,097 ± 8832		125,850 ± 20,762		76,714 ± 10,179		96,637 ± 18,260		28,824 ± 7229		27,349 ± 5948	
Filtered	130,527 ± 18,037	127,603 ± 18,531	75,499 ± 8759	74,641 ± 8592	125,412 ± 20,590	122,370 ± 21,141	76,100 ± 10,145	74,525 ± 9761	95,644 ± 18,017	94,877 ± 17,812	28,710 ± 7203	28,634 ± 7190	27,241 ± 5936	27,121 ± 5913
Merged	108,774 ± 17,679	106,199 ± 21,042	69,738 ± 8283	60,153 ± 11,767	107,636 ± 15,354	106,290 ± 20,538	69,894 ± 9428	59,943 ± 11,884	87,468 ± 15,495	84,744 ± 14,300	27,410 ± 6933	25,586 ± 6488	25,895 ± 5658	24,088 ± 5848
Mean OUT/ASV	108,774 ± 17,679	104,562 ± 21,262	69,738 ± 8283	59,925 ± 11,608	107,636 ± 15,354	105,564 ± 20,463	69,894 ± 9428	59,498 ± 11,848	87,468 ± 15,495	84,313 ± 14,067	27,410 ± 6933	25,453 ± 6396	25,895 ± 5658	23,924 ± 5800
Minimum Library	74,762	61,411	59,937	38,563	89,194	81,401	57,013	36,259	52,511	53,995	15,782	15,900	18,870	15,684
Minimum Library/Raw	0.75	0.62	0.90	0.50	0.81	0.76	0.90	0.42	0.87	0.79	0.92	0.80	0.93	0.77
Maximum Library	130,655	124,430	86,034	76,782	138,066	143,534	87,207	80,318	113,400	110,937	47,233	44,327	39,295	36,755
Maximum Library/Raw	0.87	0.87	0.93	0.88	0.89	0.90	0.93	0.88	0.93	0.94	0.96	0.97	0.97	0.95
Mean OUTs/ASVs	1371 ± 353	82 ± 73	706 ± 165	211 ± 116	1195 ± 280	58 ± 14	707 ± 203	158 ± 18	751 ± 249	77 ± 71	620 ± 447	243 ± 162	311 ± 72	104 ± 48
Minimum OUTs/ASVs	857	28	564	124	962	38	466	118	317	24	199	44	184	46
Maximum OUTs/ASVs	2103	271	1107	505	1837	78	1205	179	1359	321	1805	555	459	201

## Data Availability

The original contributions presented in this study are included in the article. Further inquiries can be directed to the corresponding author.
